# Single-cell transcriptomic analysis reveals a decrease in the frequency of macrophage-RGS1^high^ subsets in patients with osteoarticular tuberculosis

**DOI:** 10.1186/s10020-024-00886-9

**Published:** 2024-08-10

**Authors:** Ying Jiang, Xinqiang Zhang, Bo Wang, Liping Tang, Xin Liu, Xiudong Ding, Yueming Dong, Hong Lei, Di Wang, Huicheng Feng

**Affiliations:** 1 The Eighth Medical Center of Chinese PLA General Hospital, Beijing, 100091 People’s Republic of China; 2 Emergency Department, Chengde Central Hospital, Chengde, 067000 Hebei People’s Republic of China; 3 Institute of Immunology and Molecular Medicine, Jining Medical University, Jining, China

**Keywords:** Osteoarticular tuberculosis, scRNA, Biomarker, Macrophages, Ferroptosis

## Abstract

**Background:**

Cell subsets differentially modulate host immune responses to *Mycobacterium tuberculosis* (MTB) infection. However, the nature and functions of these subsets against osteoarticular tuberculosis (OTB) are unclear. Here, we aimed to understand the phenotypes and functions of immune cell subsets in patients with OTB using single-cell RNA sequencing (scRNA-Seq).

**Methods:**

Pathological and healthy adjacent tissues were isolated from patients with OTB and subjected to scRNA-Seq. Unsupervised clustering of cells was performed based on gene expression profiles, and uniform manifold approximation and projection was used for clustering visualization.

**Results:**

Thirteen cell subsets were identified in OTB tissues. scRNA-seq datasets of patients and healthy controls (HCs) showed that infection changed the frequency of immune cell subsets in OTB tissues. Myeloid cell examination revealed nine subsets. The frequency of macrophage-RGS1^high^ subsets decreased in OTB tissues; this increased MTB susceptibility in an SLC7A11/ferroptosis-dependent manner. Immunohistochemistry assays and flow cytometry for patients with OTB and osteoarticular bacterial infection (OBI) and HCs verified that the frequency of macrophage-RGS1^high^ subset decreased in OTB tissues and blood samples, thereby distinguishing patients with OTB from HCs and patients with OBI.

**Conclusion:**

The macrophage-RGS1^high^ subset levels were decreased in patients with OTB, and would be up-regulated after effective treatment. Therefore, the clinical significance of this study is to discover that macrophage-RGS1^high^ subset may serve as a potential biomarker for OTB diagnosis and treatment efficacy monitoring.

**Supplementary Information:**

The online version contains supplementary material available at 10.1186/s10020-024-00886-9.

## Introduction

Extra-pulmonary tuberculosis (EPTB) accounts for 15–20% of all *Mycobacterium tuberculosis* (MTB) infections. However, the rates of diagnosis and treatment of EPTB are considerably lower than those of pulmonary tuberculosis (PTB) because no reliable diagnostic markers are available (Sharma et al. 2021). Furthermore, osteoarticular tuberculosis (OTB) accounts for 10–18% of all EPTB cases (Khan et al. 2021). OTB symptoms are often non-specific, presenting long before suspected diagnosis. Despite advances in examination methods, OTB diagnosis remains difficult and time-consuming. Diagnosis typically relies on bacteriological and/or histopathological confirmation of MTB in the affected area (Jia et al. 2013). Rapid diagnosis is crucial for early initiation of anti-OTB chemotherapy, which can reduce disability and functional impairment.

Whole-genome sequencing technology can be used to identify specific markers of PTB and clarify the immune mechanisms (Singhania et al. 2021; Cai et al. 2020). Compared to bulk RNA sequencing (RNA-Seq), which provides only the average expression signal for millions of cells, single-cell RNA sequencing (scRNA-Seq) allows simultaneous analysis of > 10,000 single-cell transcriptomes and characterization of novel cell subsets (Cai et al. 2020). scRNA-Seq can reliably identify closely related cell populations, reveal changes that render each individual cell type unique, and elucidate the heterogeneity of gene expression patterns in peripheral blood cell populations in healthy individuals and patients (Chen et al. 2021a, b, c). Changes in subpopulation distribution and heterogeneity of blood immune cells in pulmonary tuberculosis have been detected using scRNA-Seq (Cai et al. 2020). However, scRNA-Seq analysis of OTB cases has not been reported. In this study, we subjected pathological tissues (PTs) and healthy adjacent tissues (ATs) from patients to scRNA-Seq for delineating the transcriptomic profiles of individual immune cell subsets.

## Methods

### Clinical samples

Tissue and blood samples from patients who had visited the Eighth Medical Center of the Chinese PLA General Hospital (Beijing, China) during 2020–2022 were used in the study. The diagnostic criteria for OTB (Wang et al. 2020) were as follows: patients with (i) typical symptoms of tuberculosis infection, including mild fever, night sweats, weight loss, and fatigue; (ii) MTB antibody positivity; (iii) tuberculosis granuloma; and (iv) typical features of OTB on imaging. Patients with other immune and neoplastic diseases or HIV infection were excluded. The first cohort comprised three patients with OTB. The PTs and ATs (distance from PT lesion: 3 cm) of each patient were divided into two parts. One part was used for 10 × genomics scRNA-Seq and the other was used for subsequent analysis. Supplementary Table 1 provides detailed information on scRNA-Seq analyses of these samples and the clinical features of donors. The second cohort comprised 27 patients with OTB (including the 3 patients in the first cohort); their PTs and ATs were used for specific analyses. Age- and sex-matched healthy volunteers (n = 27) and patients with osteoarticular bacterial infections (OBI; n = 27) were included as controls. Blood samples from all included participants were collected on the 1st day of their enrolment. The clinical features of all included participants in the second cohort are shown in Supplementary Table 2. All patients with OTB were examined using CD68 staining, tuberculosis DNA typing, and culturing.

This study was approved by the Eighth Medical Center of the Chinese PLA General Hospital, Beijing, China (ref no. 309202201041537). All experiments and sampling were conducted in accordance with the approved institutional guidelines for ethical and biosafety protocols. Written informed consent was obtained from all participants.

### Single-cell RNA sequencing

Details of scRNA-Seq and subsequent bioinformatic analysis, which were performed as described previously (Macosko et al. 2015; Satija et al. 2015; Butler et al. 2018; Zhang et al. 2020), are presented in the Supplementary Text.

### Molecular validation

Multiple molecular methods were used for subsequent validation, as described previously (Wang et al. 2022; Liu et al. 2014; Chen et al. 2021a, b, c). Detailed protocols are described in the Supplementary Text.

### In vivo osteoarticular tuberculosis model

The in vivo osteoarticular tuberculosis model was constructed according to a previous report (Zhou et al. 2018). New Zealand rabbits were randomly divided into two groups. An artificial bone defect was created on the femoral condyle and contaminated with 0.1 mL a tubercle bacilli suspension containing 10^6^ colony forming units (CFUs). After 7 days, the rabbits were treated with an RGS1 inhibitor (CCG-63808, 0.05 mg/kg; Sigma-Aldrich, St. Louis, MO, USA) via intraperitoneal injection every 2 days. After 4 weeks, blood was collected from the rabbits, which were subsequently sacrificed. One part of the infected tissue was used for hematoxylin and eosin (H&E) staining or cytokine measurement and the other part was homogenized and plated on Middlebrook 7H10 agar medium (262,710; BD Biosciences) to determine the CFUs.

### General statistical analysis

Data are expressed as mean ± standard deviation. All experiments were performed in no less than triplicate. All statistical analyses were performed using SPSS 24.0 software (SPSS) and GraphPad (Prism 8.0, GraphPad). Descriptive data were summarized as mean (SD). The difference between two group were compared using Student's t-test for continuous variables and using Pearson's Chi-square test for discrete variables. If any continuous variables were non-parametric, Mann–Whitney U-test was employed. Statistical significance was set at p < 0.05. Receiver operating characteristic (ROC) curves were used to evaluate the performance of the diagnostic test.

## Results

### scRNA-Seq analysis resolved major cell types in human OTB

**Fig. 1:**
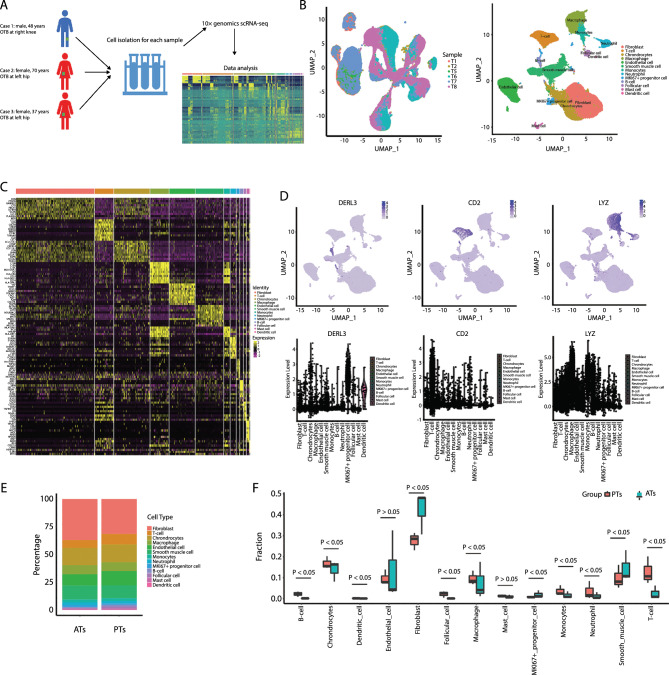
Single-cell transcriptional profiling of OTB PTs and ATs in the first cohort. **A** Schematic representation of the experimental workflow for defining and comparing OTB PTs and ATs. **B** Left: UMAP plot of cells from T1, T2, T5, T6, T7, and T8 samples. Right: UMAP of a single-cell profile, with each cell color-coded for its associated cell type. **C)** Heat map showing the expression of marker genes in the indicated cell types. **D** Top: UMAP plot showing the expression of marker genes for macrophages/monocytes, T cells, and B cells defined above each panel. Bottom: The expression of known T&NK cell type-discriminating genes. **E** Proportion of the fraction of cells for the 13 cell types in OTB PTs and ATs. **F** Box plot showing the fraction of the 13 cell types in OTB PTs and ATs. *PT* OTB pathological tissue, *AT* OTB adjacent tissue

To determine the cellular composition of human OTB, we conducted scRNA-Seq analysis (10 × Genomics Chromium System) of cells on the PTs and ATs from three patients with OTB in the first cohort (Fig. 1A). Detailed clinical information on the collected samples is provided in Supplementary Table 1. The three patients were diagnosed with OTB based on their medical history, imaging results (Supplementary Fig. 1A–C), bacteriological examinations, and histopathological examinations (Supplementary Fig. 2A–C). Quality control statistical results of the filtered data are shown in Supplementary Table 3. Sequencing quality distribution and base distribution checks showed that the filtered data were qualified and could be used for subsequent analysis (Supplementary Fig. 3A, B).

The UMAP plot revealed a high clustering of cells across the OTB PTs and ATs from each patient (Fig. 1B, left). SingleR was used for automatic annotation of major cell types, and it revealed 13 distinct cell clusters across all included participants (Fig. 1B right, C). As key immune clusters, macrophages and monocytes expressed LYZ, T cells expressed CD2 expression, and B cells expressed DERL3 (Fig. 1D), which are the known markers for the indicated cell types. Also, we identified a series of transcripts that are specifically expressed in specific immune cell types: C1QC, AIF1, CCL3, and HLA-DQA1 for macrophages and monocytes (Supplementary Fig. 4A); CCL5, GZMK, IL-7R, and TUBA4A for T cells (Supplementary Fig. 4B); and FKBP11 and MZB1 for B cells (Supplementary Fig. 4C). Specific expressed transcripts for other cell types were also identified (Supplementary Fig. 5A, B). Based on these annotations, we calculated the proportion of each cell type in PTs group and ATs group (Fig. 1E, F). Fibroblasts represented the largest fraction of all annotated cells. Macrophages and monocytes accounted for the greatest proportion of infiltrating immune cells, followed by T and B cells (Fig. 1E). Small clusters of dendritic cells (DCs) and neutrophils were also observed. T cells and macrophages showed a significantly higher frequency in the PT group than in the AT group (Fig. 1F; p < 0.05). B cells were not annotated in ATs. Only a small cluster of B cells was annotated in PTs, suggesting that B cells might play a secondary role in anti-MTB infection, which aligns with the findings of a previous study (Cai et al. 2020). Notably, the number of fibroblasts in the PT group was markedly lower than that in the AT group. Further research is needed to elucidate the molecular mechanisms underlying this phenotype (Fig. 1F).

### scRNA-Seq identified eight T and natural killer cell subsets and three B cell subsets

**Fig. 2:**
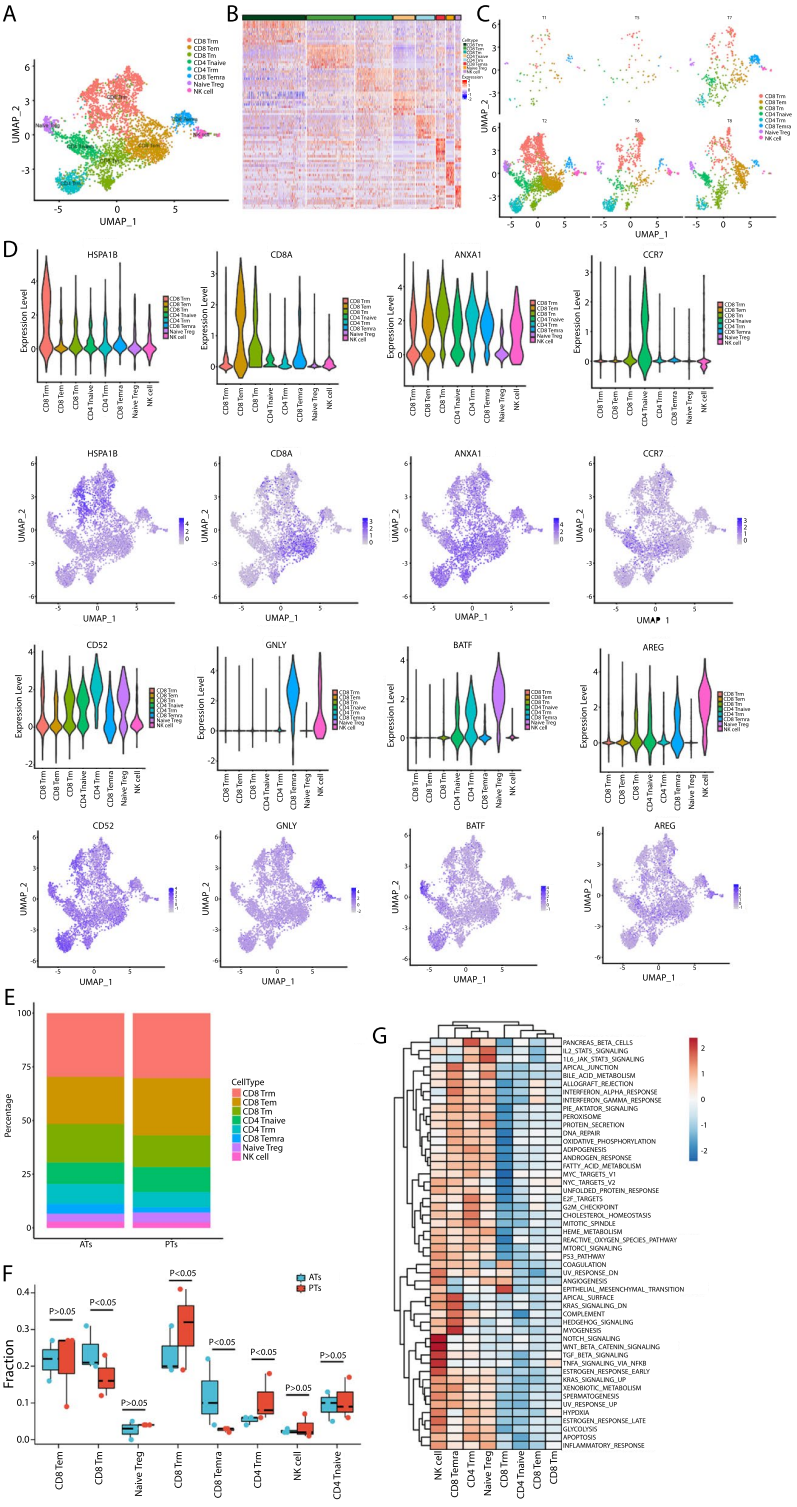
T&NK cell clusters in OTB PTs and ATs in the first cohort. **A** UMAP of a single-cell profile with each cell color-coded for its associated T&NK cell type. **B** Heat map showing the expression of marker genes in the indicated cell types. **C** UMAP plot of T&NK cell types from T1, T2, T5, T6, T7, and T8 samples. **D** Top: UMAP plot showing the expression of marker genes for the T&NK subsets; bottom: The expression of known T&NK cell type-discriminating genes. **E** Proportion of the fraction of T&NK subsets. **F** Box plot showing the fraction of T&NK subsets in OTB PTs and ATs. **G** Differences in pathway activities scored per cell analyzed using GSVA between the different T&NK subsets. *PT* OTB pathological tissue, *AT* OTB adjacent tissue

T and natural killer (T&NK) cells play a critical role in controlling MTB infection in patients with MTB (Cai et al. 2020). Therefore, we further explore the phenotypic differences in T&NK between PTs group and ATs group. First, we identified eight T&NK cell subsets based on singleR automatic annotation (Fig. 2A and B). The UMAP analysis revealed that the T&NK cell subsets in PTs presented strong heterogeneity compared with those in ATs, and the clustering was similar among different patients, indicating that they exhibited high clustering by source rather than by individuals (Fig. 1C). Among the 8 subsets, 4 were CD8 +T cell subsets, including CD8 +Trm, CD8 +Tem, CD8 +Tm, and CD8 +Temra; 2 were CD4 +T cell subsets including CD4 +Tnaive and CD4 +Trm. The other two clusters were naïve Treg cells and NK subset. HSPA1B (CD8 +Trm), CD8A (CD8 +Tem), ANXA1 (CD8 +Tm), CCR7 (CD4 +Tnaive), CD52 (D4 +Trm), BATF (naïve Treg) and AREG (NK) were found to have the most specific expression characteristics in their respective specific cell subtypes, and their expression distributions were shown in Fig. 2D.

The proportions of the eight T&NK cell subsets are shown in Fig. 2E, F. CD8 +Trm accounted for the highest proportion (29.72%), followed by CD8 +Tem (22.63%), CD8 +Tm (17.46%), and CD4 +Tnaive (10.25%). The proportions of these subsets varied between the PTs and ATs. Predictably, most subtypes were more prevalent in PTs than in ATs. Specifically, CD8 +Tm was lower in PTs than in ATs (Fig. 2F). GSVA revealed that CD8 +Tm cells exhibited a low rate of nearly all signaling, suggesting that these cells were not activated (Fig. 2G).

Although the cluster of B cells was small, we identified three B cell subsets (Supplementary Fig. 6A–C), including memory B cells highly expressed HSPA1B, IGA+plasma highly expressed IGHA2, and IGG+plasma highly expressed (Supplementary Fig. 6D). The clustering and proportion of the three cell subtypes were similar, both by source and individual (Supplementary Fig. 6C, E). GSVA revealed the specific signaling pathway of the three B cell subtypes, which might be valuable for future analyses (Supplementary Fig. 6F).

### scRNA-Seq identified nine macrophage/monocyte subsets

**Fig. 3:**
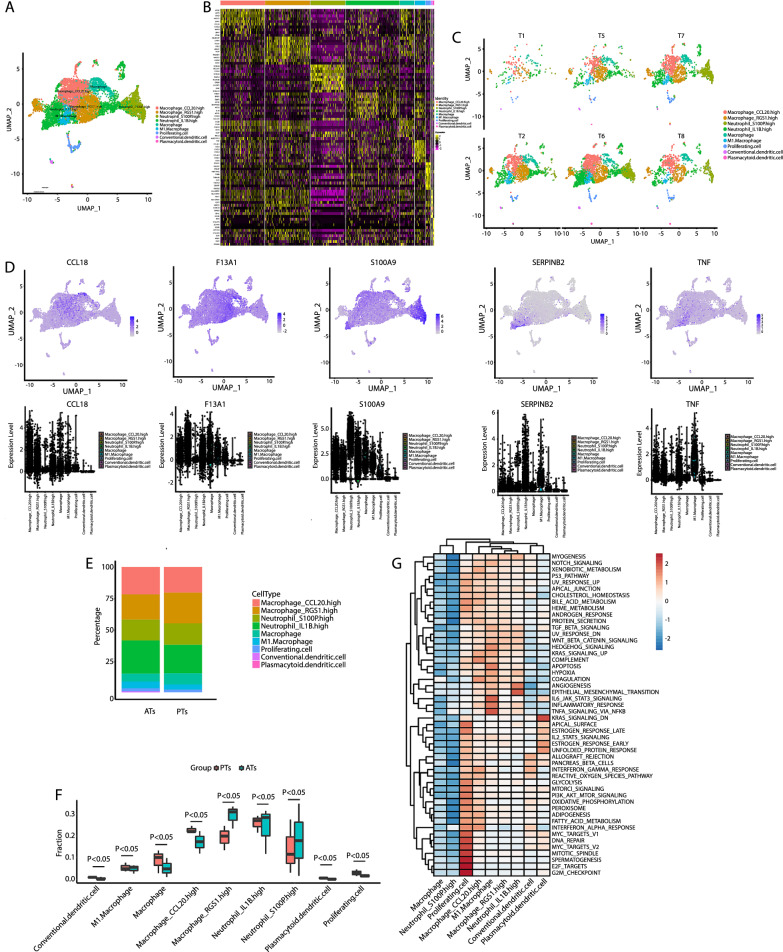
Myeloid clusters in OTB PTs and ATs in the first cohort. **A** UMAP of a single-cell profile, with each cell color-coded for its associated myeloid cell type. **B** Heat map showing the expression of marker genes in the indicated cell types. **C** UMAP plot of myeloid cell types from T1, T2, T5, T6, T7, and T8 samples. **D** Top: UMAP plot showing the expression of marker genes for the myeloid subsets; bottom: The expression of known myeloid type-discriminating genes. **E** Proportion of the fraction of myeloid subsets. **F** Box plot showing the fraction of nine myeloid cell types in OTB PTs and ATs. **G** Differences in pathway activities scored per cell via GSVA between the different myeloid subsets. *PT* OTB pathological tissue, *AT* OTB adjacent tissue

MTB infection induces the accumulation of myeloid cells that express high levels of inflammatory markers (Cai et al. 2020). This study also showed that myeloid cells, including macrophages, monocytes, neutrophils, and DCs, were the largest immune cell group in OTB tissues. Therefore, the annotation and analysis of myeloid cells were the main focus of this study. Using several recently reported markers (Chen et al. 2021a, b, c; Zou et al. 2021), we identified nine clusters in the myeloid lineage expressing specific marker genes (Fig. 3A, B): 4 clusters of macrophages (macrophages-CCL20^high^, macrophage-RGS1^high^, M1 macrophage, and all other macrophages), 2 clusters of neutrophils (neutrophils-S100P^high^ and neutrophils-IL1B^high^), 2 clusters of DCs (conventional DCs and plasma cytoid DCs), and 1 cluster of proliferating cells. The uniform manifold approximation analysis of samples revealed that the myeloid cell subsets in PTs presented strong heterogeneity compared with those in ATs, and the clustering was similar among different patients, indicating that they exhibited high clustering by source rather than by individuals (Fig. 3C). The clusters of these subsets and their markers are shown in Fig. 3D. In addition to CCL20, macrophages-CCL20^high^ were also enriched for CCL18. Macrophages-RGS1^high^ expressed high levels of F13A1, which was new for this subset. Neutrophils-S100P^high^ was enriched for S100A9 and neutrophils-IL1B^high^ expressed high levels of SERPINB2. M1 macrophages, which are recruited during MTB infection (Nguyen et al. 2021), were confirmed to express high levels of TNF l (Ren et al. 2021). The proportion of all myeloid cells in PTs and ATs is shown in Fig. 3E. Macrophages-CCL20^high^ and macrophages-RGS1^high^ accounted for the highest proportion of macrophages (Fig. 3E). The frequency of most myeloid cells was markedly higher in the PT group than in the AT group (Fig. 3F). Interestingly, macrophage-RGS1^high^ frequency decreased in the PT group, warranting further exploration (Fig. 3F). GSVA showed that macrophages-RGS1^high^ downregulated various inflammatory signaling pathways compared to M1 polarized macrophages (Fig. 3G).

**Fig. 4:**
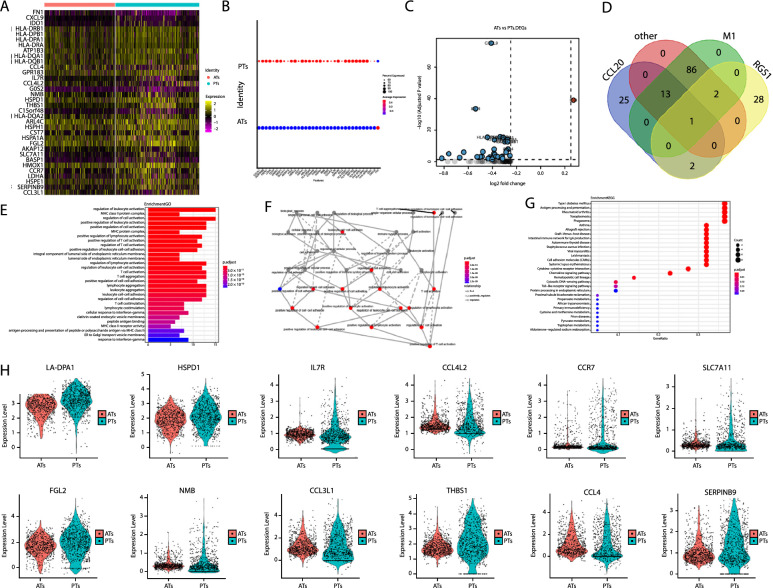
Differentially expressed genes in macrophage-RGS1^high^ subsets between PTs and ATs in the first cohort. **A** Heat maps. **B** Bubble Chart. **C** Volcano plot displaying the upregulated and downregulated genes in macrophage-RGS1^high^ subsets between in OTB PTs and ATs. **D** Venn plot displaying the overlapped upregulated and downregulated genes among macrophage-RGS1^high^ subsets, macrophages-CCL20^high^ subsets, and M1 macrophages subsets. **E** GO term column of the genes described in (**A**‒**C**). **F** Directed acyclic graphs (DAGs) for the GO term described in (E). **G** KEGG pathway term scatter diagram of the genes described in (**A**‒**C**). **H** Violin plots showing the top differentially expressed target genes in macrophage-RGS1^high^ subsets between OTB PTs and ATs

We determined the differentially expressed transcripts in all myeloid cells between the PT and AT groups to study their role in patients with OTB. The data obtained from this analysis were extensive; therefore, we have provided only the findings for all annotated myeloid cells (data for other cells are not shown), including heat map, volcano plot, bubble map, Gene Ontology (GO) analysis results, and Kyoto Encyclopedia of Genes and Genomes (KEGG) analysis results (Fig. 4A–G). We focused on the macrophage-RGS1^high^ subsets because they are specially distributed in OTB tissues. Furthermore, the differentially expressed genes (DEGs) in the macrophage-RGS1^high^ subsets were different from other subsets, such as macrophages-CCL20^high^ and M1 macrophages (Fig. 4D), suggesting a specific role of the macrophage-RGS1^high^ subsets against OTB. Among the top differentially expressed target genes in the macrophage-RGS1^high^ subsets (Fig. 4H), SLC7A11, the key inhibitor of ferroptosis, was highly expressed in the PTs group.

### RGS1 increased both ferroptosis and the intracellular killing ability of macrophage

**Fig. 5:**
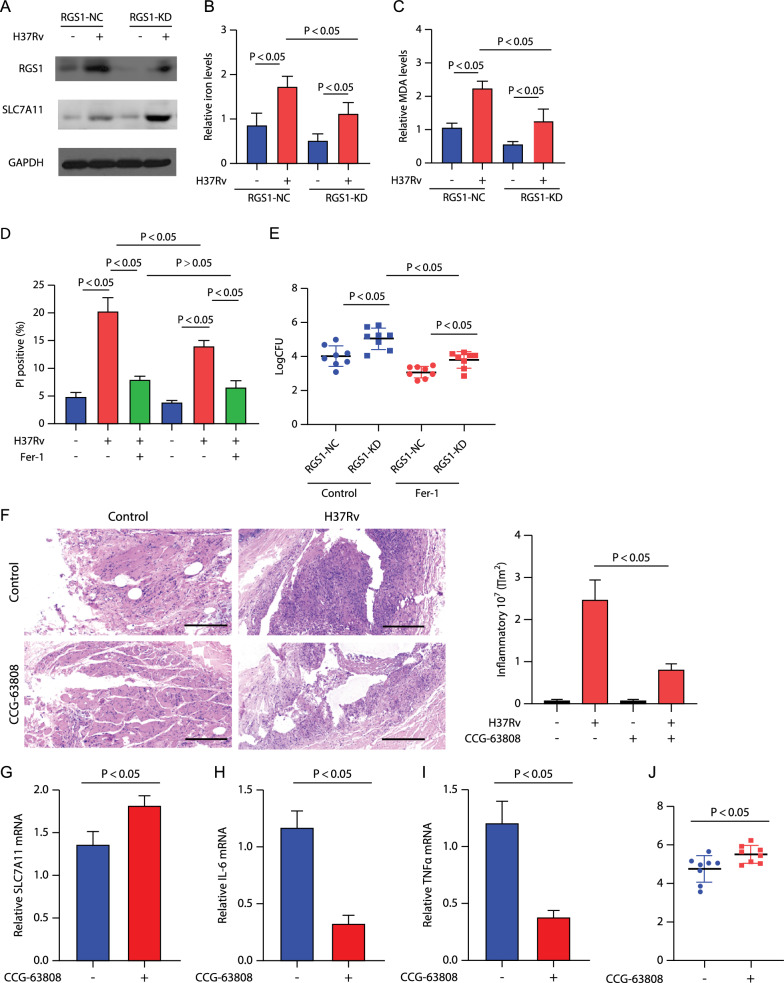
Function of RGS1 on the MTB infection in vitro and in vivo. **A**–**C** THP-1-derived macrophages were transfected with RGS1 siRNA (RGS1-KD) and the negative control (RGS1-NC) for 24 h, and then challenged with H37Rv (MOI at 10:1) for 24 h. **A** RGS1 and SLC7A11 levels were determined using western blot assay. **B** Relative iron levels were determined using an iron assay kit. **C** Relative MDA levels were determined using a lipid peroxidation assay kit. **D** THP-1-derived macrophages subjected to RGS1-NC or RGS1-KD treatment were challenged with H37Rv (MOI at 10:1) for 24 h, with or without fer-1 pre-treatment at 2 μM; then, cell necrosis was determined using PI staining followed by flow cytometry analysis. **E** THP-1-derived macrophages subjected to RGS1-NC or RGS1-KD treatment were challenged with H37Rv (MOI at 10:1) for 24 h, with or without fer-1 pre-treatment at 2 μM; then, CFUs were quantified in supernatants pooled with cell lysates. **F–J** OTB rabbit models were treated with or without the RGS1 inhibitor CCG-63808 at 0.05 mg/kg via intraperitoneal injection every 2 days. **F** Histopathology of the tissues was assessed via H&E staining. Left: Representative field of the PT tissues; Right: inflammatory areas were calculated from the PT tissues. **G–I** Relative (**G**) SLC7A11 mRNA levels; (H) TNFα mRNA levels, and (I) IL-6 mRNA levels in the PTs. **J** MTB CFUs in PTs were determined

As the above results suggested the importance of RGS1 in the function of macrophages against MTB, we transfected RGS1 small interfering RNA (siRNA) (named RGS1-KD) into cultured THP-1-derived macrophages, followed by H37Rv infection at a multiplicity of infection (MOI) of 10 for 24 h. Western blotting demonstrated that H37Rv infection could significantly enhance RGS1 expression, and RGS1 expression was down-regulated under RGS1-KD treatment (Fig. 5A). Meanwhile, we confirmed that the SLC7A11 level was also enhanced in MTB infected macrophages. Interestingly, the RGS1-KD treatment induced the SLC7A11 expressions in both H37Rv infected and un-infected macrophages, indicating that RGS1 inhibited the SLC7A11 expressions (Fig. 5A).

As we know, SLC7A11 is a negative marker of ferroptosis (Koppula et al. 2021). Therefore, we speculated that by SLC7A11 down-regulation, RGS1 might induce ferroptosis of macrophages. To verify our hypothesis, RGS1-KD-treated and control macrophages were challenged with H37Rv at an MOI of 10 for 24 h. We found that the ferroptosis markers, such as the relative iron and MDA levels were increased by H37Rv infection, and their levels were lower under RGS1-KD treatment (Fig. 5B, C). H37Rv infection could induce cell necrosis, and the level was lower under RGS1-KD treatment. Treatment with Fer-1 (a ferroptosis inhibitor) decreased the cell necrosis rate in both RGS1-KD-treated and control macrophages and could eliminate the difference between these groups (Fig. 5D). These results confirm our speculation that RGS1 could induce the ferroptosis of macrophages.

Ferroptosis was reported to promote bacterial dissemination and tissue damage under MTB infection (Amaral et al 2019). Therefore, we next explored the regulatory role of RGS1 against MTB infection. RGS1-KD-treated and control macrophages were challenged with H37Rv at an MOI of 10 for 24 h. Interestingly, the total survival rate of bacteria was significantly higher in the RGS1-KD group than in the control, whereas Fer-1 could decrease the bacterial load (Fig. 5E). These results indicated that RGS1 might promote ferroptosis (bacterial dissemination and tissue damage promoter) as well as MTB killing simultaneously.

We next assessed the effects of RGS1 in the OTB rabbit model. The rabbits were infected with H37Rv to construct an OTB model, with or without CCG-63808 (RGS1 inhibitor) treatment, for 4 weeks. We successfully detected H37Rv in each infected tissue, indicating that an infection model was successfully constructed. Histological examination (Fig. 5F) and qRT-PCR assay confirmed that the effect of RGS1 inhibition was associated with reduced tissues inflammatory damage, which was reflected by inflammatory area, inflammatory factor levels, and enhanced SLC7A11 expressions (Fig. 5G–I). And, measurement of MTB CFU in the PTs of the CCG-63808-treated OTB rabbit revealed a marked and highly significant enhancement in bacterial load, compared with that in the untreated controls (Fig. 5J). These results indicated that RGS1 can promote both inflammatory tissue damage and sterilization in vivo.

### Validation of low frequency of macrophage-RGS1^high^ subsets in human OTB tissues

**Fig. 6:**
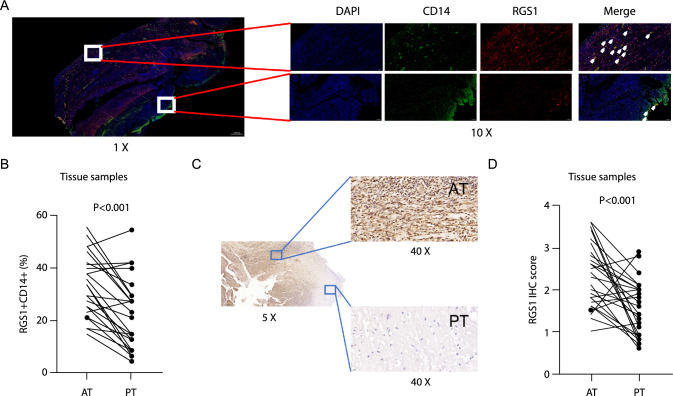
Difference in macrophage-RGS1^high^ subsets between PTs and ATs in patients with OTB in the second cohort. **A**, **B** RGS1 (red) and CD14 (green) in individual and merged channels were determined using IF in the PTs and ATs from patients with OTB (n = 27) in the second cohort. **A** Representative IF field of vision. **V** Quantification of merge counting (CD14 +RGS1 +) in the PTs and ATs from patients with OTB (n = 27) in the second cohort. **C**, **D** RGS1 staining in the PTs and ATs from patients with OTB (n = 27) in the second cohort was determined using IHC. **C** Representative immunohistochemical field of vision. **D** Quantification of RGS1 staining in the PTs and ATs from patients with OTB (n = 27) in the second cohort

Next, clinical samples from the second cohort were used to assess the effects of macrophage-RGS1^high^ subsets on patients with OTB. First, immunofluorescence analysis of CD14 (macrophage marker) and RGS1 was performed. Cells co-localizing with RGS1 and CD14 were macrophage-RGS1^high^ subsets. The expression of macrophage-RGS1^high^ subsets was mainly downregulated in PTs compared with that in the AT regions (Fig. 6A, B; p < 0.05). RGS1 was also expressed in cells other than macrophages. Overall, RGS1 expression was significantly lower in PTs, as confirmed by the results of the IHC assay (Fig. 6C, D; p < 0.05). These results are in accordance with the scRNA data.

### Low frequency of macrophage-RGS1^high^ subsets in blood samples differentiated patients with OTB and those with OBI

**Fig. 7:**
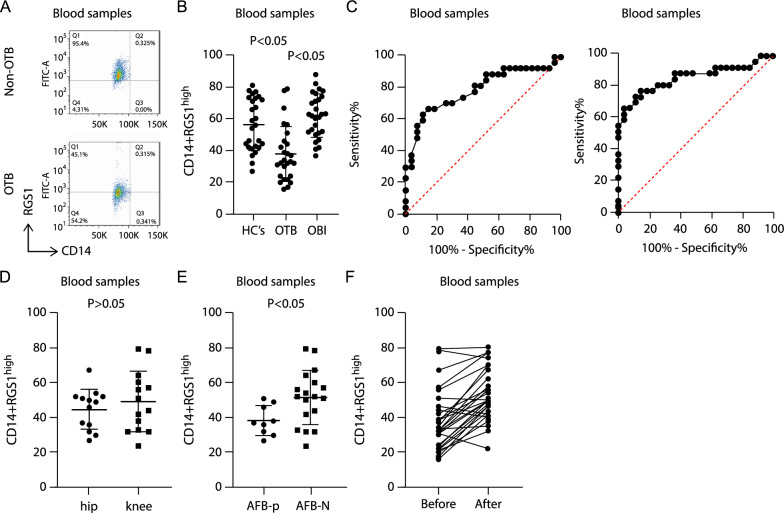
Low macrophage-RGS1^high^ subset frequency differentiates patients with OTB from HCs and patients with OBI. Macrophage-RGS1^high^ subsets were determined using flow cytometry analysis in blood samples in the second cohort. **A** Representative scatter diagram of flow cytometry analysis. **B** Comparison of the frequency of macrophage-RGS1^high^ subsets in blood samples among patients with OTB, patients with OBI, and HCs. **C** Left: ROC curve for macrophage-RGS1^high^ subsets to distinguish patients with OTB from HCs; right: ROC curve for macrophage-RGS1^high^ subsets to distinguish patients with OTB from those with OBI. **D** Comparison of the frequency of macrophage-RGS1^high^ subsets in blood samples from 13 hip infection cases and 14 knee infection cases. **E** Comparison of the frequency of macrophage-RGS1^high^ subsets in blood samples from 9 AFB+cases and 18 AFB- cases. **F** Comparison of the frequency of macrophage-RGS1^high^ subsets in blood samples from 27 patients with OTB before and after 8 weeks of anti-MTB treatment. *OTB* osteoarticular tuberculosis, *OBI* osteoarticular bacterial infection, *HC* healthy controls

We determined the phenotypes of macrophage-RGS1^high^ subset in the blood of patients with OTB using flow cytometry to explore whether this subset could act as a potential biomarker. The frequency of macrophage-RGS1^high^ subsets in blood samples was also lower in individuals with OTB than in HCs (Fig. 7A, B). Meanwhile, the frequency of macrophage-RGS1^high^ subsets appeared to be slightly higher in patients with OBI than in HCs, although the difference was not significant (Fig. 7B). The ROC curve analyses of patients with OTB vs. HCs and patients with OTB vs. those with OBI indicated that the macrophage-RGS1^high^ subset could serve as a valuable biomarker, with the area under the curve being 0.7894 (Fig. 7c, left; OTB vs. HCs, p = 0.003) and 0.8587 (Fig. 7C, right; OTB vs. OBI, p < 0.001), respectively. The macrophage-RGS1^high^ subset frequency in blood samples did not vary significantly between hip OTB cases and knee OTB cases (Fig. 7D; p > 0.05). Among all these patients with OTB, compared to acid-fast bacilli (AFB)-negative cases, AFB-positive cases showed significantly low macrophage-RGS1^high^ subset frequency in tissue samples (Fig. 7E; p < 0.05). We further analyzed the frequency of the macrophage-RGS1^high^ subset in blood samples after anti-OTB treatment. The macrophage-RGS1^high^ subset frequency increased 8 weeks after initiating treatment (Fig. 7F). These results indicate that macrophage-RGS1^high^ is useful for differentiating patients with OTB from HCs and patients with OBI and might help indicate the treatment outcome of OTB.

## Discussion

Current diagnostic methods for OTB include radiological tests combined with histopathological techniques, and microbiological results. However, these methods all have shortcomings. The radiological tests and histopathological tests lack sensitivity and specificity to provide an early diagnosis. For microbiological tests, clinical specimens are usually assessed using AFB smear, which is the fastest diagnostic test, but only 20–40% of clinical specimens test positive. MTB culturing also lacks sensitivity and takes a long time to obtain results (Jia et al. 2013). In recent years several commercial nucleic acid amplification tests, like Mycobacterial PCR and gene Xpert, have been developed for quickly identifying pathogenic MTB, but they cannot determine the activity of the pathogen (Fernandez-Pittol et al. 2021). Progression of the active MTB disease is almost universally regarded as a ‘failure’ of the host's immune system to control the infection. This process is usually accompanied by the molecular dysregulation of the critical balance between the immune cells and MTB (Wang et al. 2022). Therefore, identifying specific cell subsets in patients caused by MTB infection has the potential implications on the clinical diagnosis and treatment strategies. For example, Sun et al. reported that the cell population data could assist the distinguish of active tuberculosis and community-acquired pneumonia (Sun et al. 2021). Cai et al. identified that the frequency of CD3-CD7 +GZMB +in peripheral blood could be used as a novel biomarker for distinguishing PTB from latent tuberculosis infection (LTBI) (Cai et al. 2020). In the present study, we aimed to elucidate the effect of MTB infection on PTs using scRNA-Seq of OTB tissues and the adjacent HC tissues. We distinguished 13 major cell types and sub-clustered myeloid cells into nine subsets based on quantitative gene expression. The most noteworthy cell type included macrophage-RGS1^high^ subsets, because their frequency was significantly decreased in OTB tissues, whereas the frequency of other subtypes was typically increased.

RGS1 is a regulatory member of the G protein signaling family that links G protein-coupled receptors with calcium signaling. It is specifically expressed in macrophages, T cells, and B cells. RGS1 dysregulation may lead to various autoimmune diseases (Patel et al. 2015; Feng et al. 2021). Patel et al. reported that vascular inflammation condition in atherosclerosis increases RGS1 expression (Patel et al. 2015). Also, RGS1 has been indicated to be a new marker and promoting factor for CD8 +T cell exhaustion in tumors (Bai et al. 2021). RGS1 inhibits chemokine-induced lymphocyte migration because chemokine-dependent activation of G protein-coupled receptors can activate heterotrimeric G protein subunits, resulting in enhanced cell migration and adhesion (Bai et al. 2021). We further found that expressions of RGS1 across the Cancer Genome Atlas Program (TCGA) tumors were generally enhanced, compared with the normal controls (data not shown). Therefore, it can be seen that the expression patterns of RGS1 in OTBs is opposite to that in the OBIs, vast majority of inflammatory diseases or tumors, which is one of the reasons why it is suitable as a diagnostic marker for OTBs. Of course, RGS1 is down-regulated in some tumors such as bladder cancer, rectum adenocarcinoma, and thyroid carcinoma (from TCGA databank), which can interfere with its use as a biomarker for the diagnosis of OTB. Therefore, exploring a diagnostic strategy that combines RGS1 levels with existing diagnostic methods to improve the sensitivity and specificity of OTBs detection should be one of the next research directions.

In the present study, several genes related to cell adhesion were differentially expressed between PTs and ATs in macrophage-RGS1^high^ subsets, suggesting that the function of macrophage-RGS1^high^ subsets is altered by MTB infection. SLC7A11 expression was only upregulated in macrophage-RGS1^high^ subsets in PTs at the single-cell level. SLC7A11 imports cysteine for glutathione biosynthesis and antioxidant defense and is overexpressed in multiple human and immune diseases (Koppula et al. 2021). SLC7A11 overexpression partially suppresses ferroptosis, a form of regulated cell death induced by excessive lipid peroxidation (Lang et al. 2019). MTB infection has been found to induce ferroptosis (Amaral et al. 2019). In the present study, upregulated SLC7A11 expression were observed in macrophage-RGS1^high^ subsets under MTB infection, suggesting the functionality of macrophage-RGS1^high^ may be related to SLC7A11 and ferroptosis. Correspondingly, we confirmed that RGS1 can inhibit the expression of SLC7A11. However, the function of SLC7A11 and the process of ferroptosis against intracellular MTB are complex. It has been reported that MTB infection can upregulate not only ferroptosis but also the expression of the ferroptosis blocker SLC7A11 (Amaral et al. 2019). Meanwhile, in the present study, fer-1, a ferroptosis inhibitor through antioxidant effects, was found to inhibit MTB sensitivity; This finding indicates that the regulatory effect of SLC7A11 on MTB is different from that of ferroptosis. Cai et al. found that SLC7A11 mainly exerts bactericidal effect in tuberculosis through the TLR2/Akt- and p38-dependent signaling pathway, which is different from the ferroptosis pathway (Cai et al. 2016). The present study showed that the effect of RGS1 against MTB is also through the opposite direction to ferroptosis.

We also explored the correlation between macrophage-RGS1^high^ subset frequencies and clinical indicators in patients with OTB. The decrease in macrophage-RGS1^high^ subset frequency was further verified using tissue and blood samples. Considering that this result is not consistent with the in vitro and in vivo experimental results (MTB induces RGS1 expression), we speculate that there are differences in the expression of RGS1 at different stages of infection, attributable to the dynamics of the competition between MTB immune escape and host immune killing.

Further exploration demonstrated that the change in peripheral macrophage-RGS1^high^ subset frequency was sensitive and specific for discriminating patients with active OTB infection from HCs and patients with OBI. As blood is the most readily accessible sample in humans, we propose that macrophage-RGS1^high^ subsets could be used as biomarkers in patients with OTB. Moreover, 8 weeks of treatment can increase macrophage-RGS1^high^ subset frequency, suggesting that macrophage-RGS1^high^ subsets may also be used to monitor treatment outcomes.

Overall, the macrophage-RGS1^high^ subset could be used as a biomarker for distinguishing patients with OTB from patients with OBI and HCs.

This study has some significance as well as limitations. Firstly, to our best knowledge, this is the first to provide scRNA-Seq data for OTB tissues. However, we only analyzed the role of main immune cell subtypes (myeloid cells, T cells, and B cells). In fact, as shown in Fig. 1F, among all the annotated cell types, fibroblasts showed the greatest differential changes between the PTs group and the ATs group, indicating the potential function. Therefore, another future direction based on this study should be to explore the functions of more cell subtypes in the progression of OTB, which might accelerate the discovery of more biomarkers and therapeutic targets on OTB. Further, we should obtain the scRNA-Seq data on the tissues from non-OBT controls (such as healthy controls or OBI controls), in order to avoid potential biases. Secondly, the current conclusions on macrophage-RGS1^high^ subset function are based on a small-scale study. In the next step, larger-scale multicenter studies to eliminate the influence of regional differences or medical conditions are needed.

### Supplementary Information

Additional file 1: Figure 1 Imaging diagnosis results of the three patients in the first cohort. Figure 2 H&E staining and CD68 immunohistochemistry results of the OTB lesion tissue from (A) Patient 1, (B) Patient 2, and (C) Patient 3 in the first cohort. Figure 3 Quality control of single-cell sequencing data. Figure 4. Novel markers for macrophages/monocytes, T cells, and B cells and their clustering. Figure 5 Novel markers for specific myeloid cell subtypes and their clustering. Figure 6 B cell clusters in OTB PTs and ATs in the first cohort. Table 1. Detailed information of samples collected for scRNA-Seq analyses in the study. Table 2. Clinical characteristics of included participants in the second cohort. Table 3. Cell number and gene median statistics. Methods

## Data Availability

The datasets used and/or analyzed during the current study are available from the corresponding author on reasonable request.

## Abbreviations

AFB: Acid-fast bacilli
CFU: Colony forming unit
DC: Dendritic cell
DAG: Directed acyclic graph
EPTB: Extra-pulmonary tuberculosis
AT: Adjacent tissue
HC: Healthy control
H&E: Hematoxylin and eosin
MTB: *Mycobacterium tuberculosis*
NC: Negative control
OBI: Osteoarticular bacterial infection
OTB: Osteoarticular tuberculosis
PT: Pathological tissue
PTB: Pulmonary tuberculosis
ROC: Receiver operating characteristic
RNA-seq: RNA sequencing
SASP: Salazosulfapyridine
scRNA-Seq: Single-cell RNA sequencing
siRNA: Small interfering RNA
T&NK: T and natural killer
