# Supplementary material for: Single-cell transcriptomic analysis reveals a decrease in the frequency of macrophage-RGS1high subsets in patients with osteoarticular tuberculosis
Source: Mol Med. 2024 Aug 10;30:118. doi: 10.1186/s10020-024-00886-9 (PMC11316427; doi:10.1186/s10020-024-00886-9)
Supplement: Supplementary file 1 — Additional file 1: Figure 1 Imaging diagnosis results of the three patients in the first cohort. Figure 2 H&E staining and CD68 immunohistochemistry results of the OTB lesion tissue from (A) Patient 1, (B) Patient 2, and (C) Patient 3 in the first cohort. Figure 3 Quality control of single-cell sequencing data. Figure 4. Novel markers for macrophages/monocytes, T cells, and B cells and their clustering. Figure 5 Novel markers for specific myeloid cell subtypes and their clustering. Figure 6 B cell clusters in OTB PTs and ATs in the first cohort. Table 1. Detailed information of samples collected for scRNA-Seq analyses in the study. Table 2. Clinical characteristics of included participants in the second cohort. Table 3. Cell number and gene median statistics. Methods [file 10020_2024_886_MOESM1_ESM.zip › New folder/Supplementary_Table_1.docx]

**Supplementary Table 1.** Detailed information of samples collected for scRNA-Seq analyses in the study.

| **Sample** | **Corresponding patient** | **Tissue** | **Strategy** | **Sex** | **Age (years)** | **Diagnosis** |
| --- | --- | --- | --- | --- | --- | --- |
| Sample T1 | Patient 1 | OTB adjacent tissues | scRNA-seq | Female | 70 | OTB in left hip joint; diagnosis based on MRI results and clinical symptoms and confirmed using acid-fast staining |
| Sample T2 | Patient 1 | OTB pathological tissues | scRNA-seq |  |  |  |
| Sample T5 | Patient 2 | OTB adjacent tissues | scRNA-seq | Female | 37 | OTB in left hip joint; diagnosis based on MRI results and clinical symptoms and confirmed using acid-fast staining |
| Sample T6 | Patient 2 | OTB pathological tissues | scRNA-seq |  |  |  |
| Sample T7 | Patient 3 | OTB adjacent tissues | scRNA-seq | Male | 51 | OTB in left knee joint; diagnosis based on MRI results and clinical symptoms and confirmed using acid-fast staining |
| Sample T8 | Patient 3 | OTB pathological tissues | scRNA-seq |  |  |  |
