# Supplementary material for: Single-cell transcriptomic analysis reveals a decrease in the frequency of macrophage-RGS1high subsets in patients with osteoarticular tuberculosis
Source: Mol Med. 2024 Aug 10;30:118. doi: 10.1186/s10020-024-00886-9 (PMC11316427; doi:10.1186/s10020-024-00886-9)
Supplement: Supplementary file 1 — Additional file 1: Figure 1 Imaging diagnosis results of the three patients in the first cohort. Figure 2 H&E staining and CD68 immunohistochemistry results of the OTB lesion tissue from (A) Patient 1, (B) Patient 2, and (C) Patient 3 in the first cohort. Figure 3 Quality control of single-cell sequencing data. Figure 4. Novel markers for macrophages/monocytes, T cells, and B cells and their clustering. Figure 5 Novel markers for specific myeloid cell subtypes and their clustering. Figure 6 B cell clusters in OTB PTs and ATs in the first cohort. Table 1. Detailed information of samples collected for scRNA-Seq analyses in the study. Table 2. Clinical characteristics of included participants in the second cohort. Table 3. Cell number and gene median statistics. Methods [file 10020_2024_886_MOESM1_ESM.zip › New folder/Supplementary_Table_2.docx]

**Supplementary Table 2.** Clinical characteristics of included participants in the second cohort.

| **Characteristic** | **Patients with OTB (n = 27)** | **Patients with OBI (n = 27)** | **HCs (n = 27)** |
| --- | --- | --- | --- |
| Age (years) | 47.3 ± 18.4 | 45.1 ± 20.8 | 44.9 ± 17.6 |
| Sex (male/female) | 18/9 | 15/12 | 15/12 |
| Site of infection  Hip  Knee | 13  14 | 12  15 | ---  --- |
| Combined pulmonary TB | 5 | 0 | 0 |
| Combined kidney TB | 3 | 0 | 0 |
| CD68+ confirmed | 9/18 | --- | --- |
