# Supplementary material for: Single-cell transcriptomic analysis reveals a decrease in the frequency of macrophage-RGS1high subsets in patients with osteoarticular tuberculosis
Source: Mol Med. 2024 Aug 10;30:118. doi: 10.1186/s10020-024-00886-9 (PMC11316427; doi:10.1186/s10020-024-00886-9)
Supplement: Supplementary file 1 — Additional file 1: Figure 1 Imaging diagnosis results of the three patients in the first cohort. Figure 2 H&E staining and CD68 immunohistochemistry results of the OTB lesion tissue from (A) Patient 1, (B) Patient 2, and (C) Patient 3 in the first cohort. Figure 3 Quality control of single-cell sequencing data. Figure 4. Novel markers for macrophages/monocytes, T cells, and B cells and their clustering. Figure 5 Novel markers for specific myeloid cell subtypes and their clustering. Figure 6 B cell clusters in OTB PTs and ATs in the first cohort. Table 1. Detailed information of samples collected for scRNA-Seq analyses in the study. Table 2. Clinical characteristics of included participants in the second cohort. Table 3. Cell number and gene median statistics. Methods [file 10020_2024_886_MOESM1_ESM.zip › New folder/Supplementary_Table_3.docx]

**Supplementary Table 3.** Cell number and gene median statistics.

|  | **T1** | **T2** | **T5** | **T6** | **T7** | **T8** |
| --- | --- | --- | --- | --- | --- | --- |
| Estimated number of cells | 11,067 | 12,208 | 8,900 | 10,455 | 7,786 | 9,419 |
| Mean reads per cell | 64,234 | 50,369 | 66,933 | 49,459 | 67,236 | 53,445 |
| Median genes per cell | 1,494 | 1,440 | 1,958 | 2,039 | 2,337 | 2,851 |
| Fraction reads in cells | 89.4% | 8.1% | 87.9% | 82.0% | 81.2% | 84.0% |
| Total genes detected | 25,207 | 26,887 | 26,583 | 27,282 | 26,060 | 26,474 |
| Median UMI counts per cell | 3,744 | 3,753 | 5,724 | 6,744 | 7,048 | 9,664 |
