# Supplementary material for: Single-cell transcriptomic analysis reveals a decrease in the frequency of macrophage-RGS1high subsets in patients with osteoarticular tuberculosis
Source: Mol Med. 2024 Aug 10;30:118. doi: 10.1186/s10020-024-00886-9 (PMC11316427; doi:10.1186/s10020-024-00886-9)
Supplement: Supplementary file 1 — Additional file 1: Figure 1 Imaging diagnosis results of the three patients in the first cohort. Figure 2 H&E staining and CD68 immunohistochemistry results of the OTB lesion tissue from (A) Patient 1, (B) Patient 2, and (C) Patient 3 in the first cohort. Figure 3 Quality control of single-cell sequencing data. Figure 4. Novel markers for macrophages/monocytes, T cells, and B cells and their clustering. Figure 5 Novel markers for specific myeloid cell subtypes and their clustering. Figure 6 B cell clusters in OTB PTs and ATs in the first cohort. Table 1. Detailed information of samples collected for scRNA-Seq analyses in the study. Table 2. Clinical characteristics of included participants in the second cohort. Table 3. Cell number and gene median statistics. Methods [file 10020_2024_886_MOESM1_ESM.zip › New folder/Supplementary_Text-20240705.docx]

**Supplementary Methods**

**10X genomics single-cell sample processing, cDNA library preparation, and sequencing**

Single-cell RNA-Seq libraries were prepared using Chromium Single Cell 3ʹ Reagent Kits v3 (10X Genomics) according to the manufacturer’s instructions. Briefly, a tissue sample was digested into a single-cell suspension using collagenase II (cell viability ≥ 85%, and total amount ≥ 1 × 10^6^ cells/mL). The cells were captured in droplets during targeted cell recovery. After the reverse transcription step, the emulsions were broken, and barcoded complementary DNA (cDNA) was purified, followed by PCR amplification. The amplified cDNA was then used to construct the 3′ gene expression library. For gene expression library construction, 50 ng of amplified cDNA was fragmented and end-repaired, double-size selected with SPRI select beads, and sequenced on a NovaSeq platform (Illumina Inc., San Diego, CA, USA) to generate 150-bp paired-end reads. The raw sequencing data were uploaded to the National Omics Data Encyclopedia website (https://www.biosino.org/node/) and made publicly available when the article was published.

**Generation and analysis of single-cell transcriptomes**

Raw reads were demultiplexed and mapped to the reference genome using the 10X Genomics Cell Ranger pipeline (https://support.10xgenomics.com/single-cell-geneexpression/software/pipelines/latest/what-is-cell-ranger) with default parameters. All downstream single-cell analyses were performed using Cell Ranger and Seurat unless otherwise mentioned (Macosko et al. 2015; Satija et al. 2015). For each gene and cell barcode (filtered by Cell Ranger), unique molecule identifiers were counted to construct digital expression matrices. For secondary filtration by Seurat, a gene with expression in more than three cells was considered to be expressed, and each cell was required to have at least 200 expressed genes; some foreign cells were removed. The Cell Ranger count uses data in FASTQ files to perform alignment, filtering, barcode counting, and unique molecular identifier counting. It uses chromium cellular barcodes to generate feature barcode matrices by Cell Ranger count and reruns the dimensionality reduction, clustering, and gene expression algorithms using the default parameter settings. Secondary analysis of gene expression was performed using Seurat. The Seurat package was used to normalize data, reduce dimensionality for uniform manifold approximation and projection (UMAP) analysis, and cluster differential expression. Marker genes for each cluster were identified with the wilcox (Wilcoxon Rank Sum test) with default parameters via the FindAllMarkers function in Seurat. Canonical correlation analysis was used for the integrated analysis of datasets (Satija et al. 2015; Butler et al. 2018). For clustering, highly variable genes were selected, and the principal components based on these genes were used to construct a graph, which was segmented with a resolution of 0.6. Cell annotation was automatically implemented using singleR, or based on reported markers if special description were indicated.

**Bioinformatic analysis**

Gene Ontology (GO) and Kyoto Encyclopedia of Genes and Genomes (KEGG) enrichment analyses of differentially expressed gene (DEG) sets were implemented in the GOseq R and KOBAS 3.0 packages, respectively. GO terms with adjusted p-values < 0.05 were considered significantly enriched by DEGs (Zhang et al. 2020). Gene set variation analysis (GSVA)was performed on the 50 hallmark pathways annotated in the molecular signature database (Cai et al. 2020).

**Immunohistochemistry and immunofluorescence**

OTB PTs and ATs from the second cohort were used in the immunohistochemistry (IHC) and immunofluorescence (IF) assays. The IHC protocol used has been detailed in our previous report (Wang et al. 2022). An anti-RGS1 antibody (Cat# ab117077; Abcam, Cambridge, UK) was used to detect RGS1. The sections were photographed (Leica DM2000 LED Microscope; Leica Microsystems GmbH, Wetzlar, Germany), and the staining intensity was analyzed (Image-Pro Plus v6.0; Media Cybernetics Inc., Rockville, MD, USA). RGS1 expression was quantified using the mean optical density. F assay specific for CD14 (Cat# 17000-1-AP; Protein tech, Wuhan, China) and RGS1 (Cat# ab117077; Abcam, Cambridge, UK) was conducted. The sections were photographed (Nikon Eclipse Ts2 Inverted Biological Microscope; Nikon, Tokyo, Japan), and the staining intensity was analyzed (Image-Pro Plus v6.0). Images were analyzed and quantified using the inForm software (v2.3, PerkinElmer Inc.) based on an active machine learning algorithm with a pre-visual cutoff followed by a single-cell-based mean pixel fluorescence intensity to achieve accuracy. For both IHC and IF, one slide of the PTs was randomly selected, and the normalized RGS1 level was determined as “1,” whereas normalized RGS1 levels of other samples were set as the “fold change” relative to that sample.

**Flow cytometry**

OTB PTs and ATs and blood samples from patients with OTB, patients with OBI, and healthy controls (HCs) from the second cohort were used for flow cytometry analysis (Wang et al. 2022). Briefly, cells harvested from tissues or peripheral blood mononuclear cells (PBMCs) were stained with antibodies in phosphate-buffered saline (PBS) containing 0.1% (w/v) bovine serum albumin and 0.1% NaN_3_. Human BD Fc Block antibody (BD Biosciences, Franklin Lakes, NJ, USA) was used to block Fc in these samples. Flow cytometry data were acquired on a FACSCalibur (BD Biosciences) or Beckman Coulter Epics XL benchtop flow cytometer (Beckman Coulter Inc., Brea, CA, USA). Data were analyzed using FlowJo (version 10.0.6, TreeStar, Ashland, OR, USA). Polyclonal rabbit anti‑human RGS1 antibody (Cat# LS-C162570-400; LSBio, Seattle, WA, USA) and FITC-conjugated goat-anti-rabbit secondary antibodies were used for RGS1 analysis. PE-linked CD14 monoclonal antibody (61D3; Cat# 12-0149-42; Thermo Fisher Scientific, Waltham, MA, USA) was used for CD14 analysis.

***In vitro* macrophage MTB infection model**

The standard MTB H37Rv strain (ATCC 27294) was cultured in Middlebrook 7H10 agar medium before use. THP-1-derived macrophages were differentiated into macrophages according to our previous report (Liu et al. 2014). *In vitro* macrophage MTB infection model establishment and colony-forming unit (CFU) determination were performed according to our previous reports (Wang et al. 2022; Liu et al. 2014; Chen et al. 2021).

**Western blotting**

Protein extracts were prepared in RIPA lysis buffer (Cat# 89900; Thermo Fisher Scientific). Protein quantification was performed using the BCA Protein Assay Kit (Cat# 23225; Thermo Fisher Scientific). Proteins were separated using sodium dodecyl sulfate-polyacrylamide gel electrophoresis and transferred onto a polyvinylidene fluoride (PVDF) membrane (Cat# 88585; Thermo Fisher Scientific). After blocking, the blots were incubated with the primary antibody at 4°C overnight and then with a secondary antibody. The protein bands were visualized using enhanced chemiluminescence and film exposure. The primary antibodies used were xCT/SLC7A11 (Cat# 12691; Cell Signaling Technology, Danvers, MA, USA) and GAPDH (Cat# 5174; Cell Signaling Technology).

**Necrotic cell death determination**

Necrotic cell death was analyzed using propidium iodide staining (Cat# 00-6990-50; Thermo Fisher Scientific), followed by flow cytometry. Cells were fixed in cold 70% ethanol for at least 30 min at 4°C, centrifuged at 2,000 rpm for 10 min, resuspended in PBS, treated with RNase, and stained with propidium iodide (Cat# BMS500PI; Thermo Fisher Scientific). The cells were analyzed using the FACSCalibur system (BD Biosciences).
