# Supplementary figures and images for: Single-cell transcriptomic analysis reveals a decrease in the frequency of macrophage-RGS1high subsets in patients with osteoarticular tuberculosis
Source: Mol Med. 2024 Aug 10;30:118. doi: 10.1186/s10020-024-00886-9 (PMC11316427; doi:10.1186/s10020-024-00886-9)

A

Patient 1: X-ray

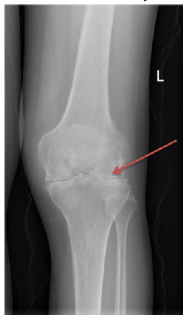

CT

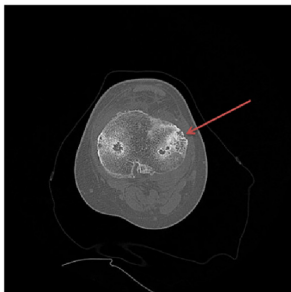

MRI

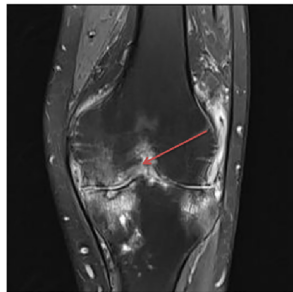

B

Patient 2: X-ray

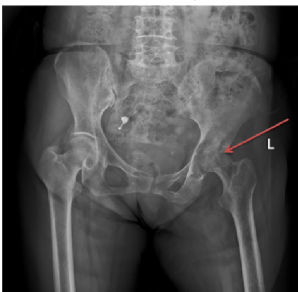

CT

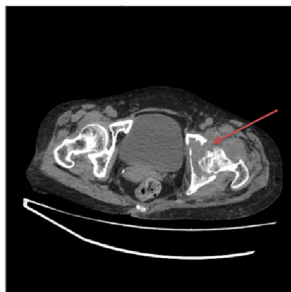

C

Patient 3: X-ray

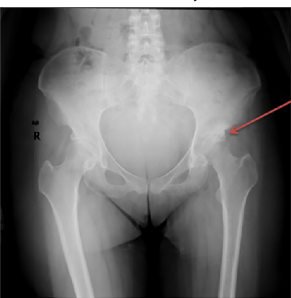

CT

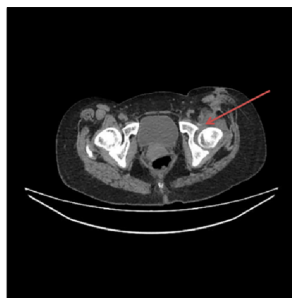

MRI

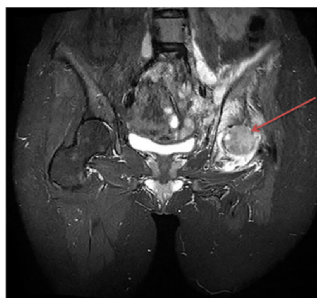

Supplement: Supplementary file 1 — Additional file 1: Figure 1 Imaging diagnosis results of the three patients in the first cohort. Figure 2 H&E staining and CD68 immunohistochemistry results of the OTB lesion tissue from (A) Patient 1, (B) Patient 2, and (C) Patient 3 in the first cohort. Figure 3 Quality control of single-cell sequencing data. Figure 4. Novel markers for macrophages/monocytes, T cells, and B cells and their clustering. Figure 5 Novel markers for specific myeloid cell subtypes and their clustering. Figure 6 B cell clusters in OTB PTs and ATs in the first cohort. Table 1. Detailed information of samples collected for scRNA-Seq analyses in the study. Table 2. Clinical characteristics of included participants in the second cohort. Table 3. Cell number and gene median statistics. Methods [file 10020_2024_886_MOESM1_ESM.zip › New folder/Sup Fig1-revised.pdf]

**A**

Patient 1: H&amp;E

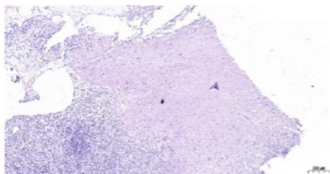

CD68+

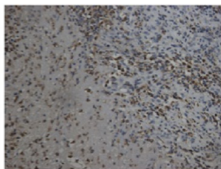**B**

Patient 2: H&amp;E

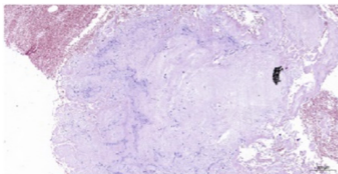

CD68+

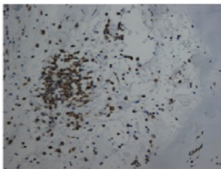**C**

Patient 3: H&amp;E

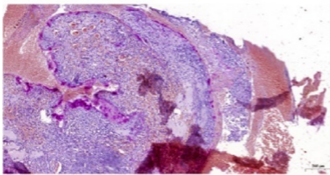

CD68+

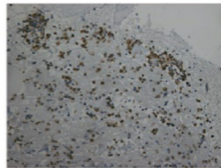

Supplement: Supplementary file 1 — Additional file 1: Figure 1 Imaging diagnosis results of the three patients in the first cohort. Figure 2 H&E staining and CD68 immunohistochemistry results of the OTB lesion tissue from (A) Patient 1, (B) Patient 2, and (C) Patient 3 in the first cohort. Figure 3 Quality control of single-cell sequencing data. Figure 4. Novel markers for macrophages/monocytes, T cells, and B cells and their clustering. Figure 5 Novel markers for specific myeloid cell subtypes and their clustering. Figure 6 B cell clusters in OTB PTs and ATs in the first cohort. Table 1. Detailed information of samples collected for scRNA-Seq analyses in the study. Table 2. Clinical characteristics of included participants in the second cohort. Table 3. Cell number and gene median statistics. Methods [file 10020_2024_886_MOESM1_ESM.zip › New folder/Sup Fig2-revised.pdf]

**A**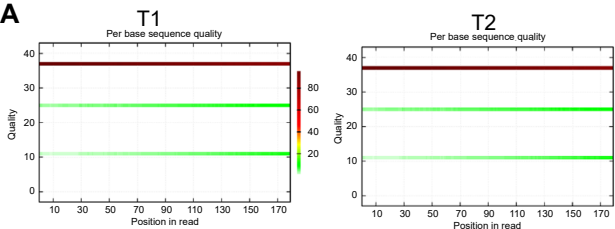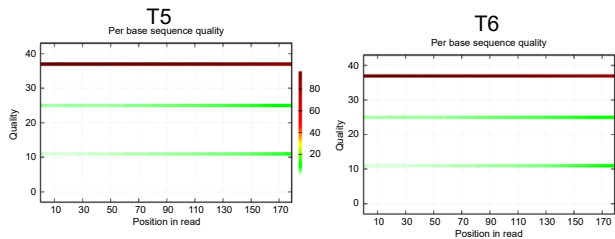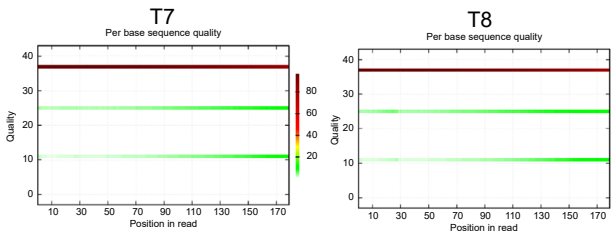**B**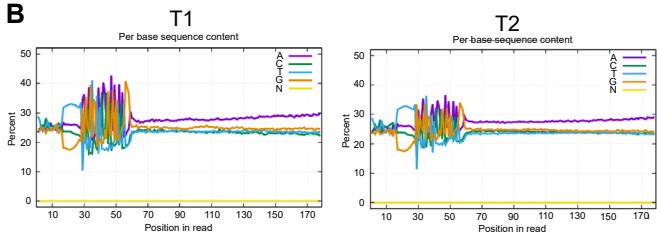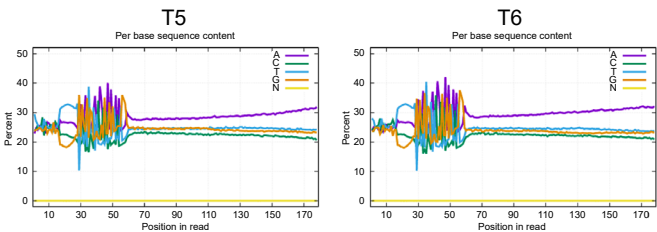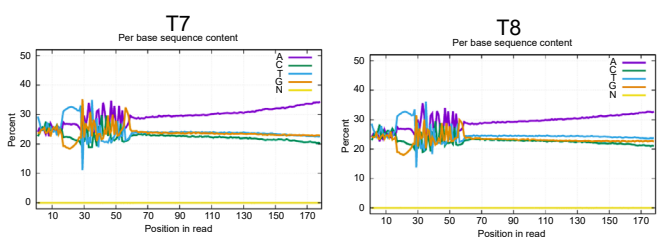

Supplement: Supplementary file 1 — Additional file 1: Figure 1 Imaging diagnosis results of the three patients in the first cohort. Figure 2 H&E staining and CD68 immunohistochemistry results of the OTB lesion tissue from (A) Patient 1, (B) Patient 2, and (C) Patient 3 in the first cohort. Figure 3 Quality control of single-cell sequencing data. Figure 4. Novel markers for macrophages/monocytes, T cells, and B cells and their clustering. Figure 5 Novel markers for specific myeloid cell subtypes and their clustering. Figure 6 B cell clusters in OTB PTs and ATs in the first cohort. Table 1. Detailed information of samples collected for scRNA-Seq analyses in the study. Table 2. Clinical characteristics of included participants in the second cohort. Table 3. Cell number and gene median statistics. Methods [file 10020_2024_886_MOESM1_ESM.zip › New folder/Sup Fig3-revised.pdf]

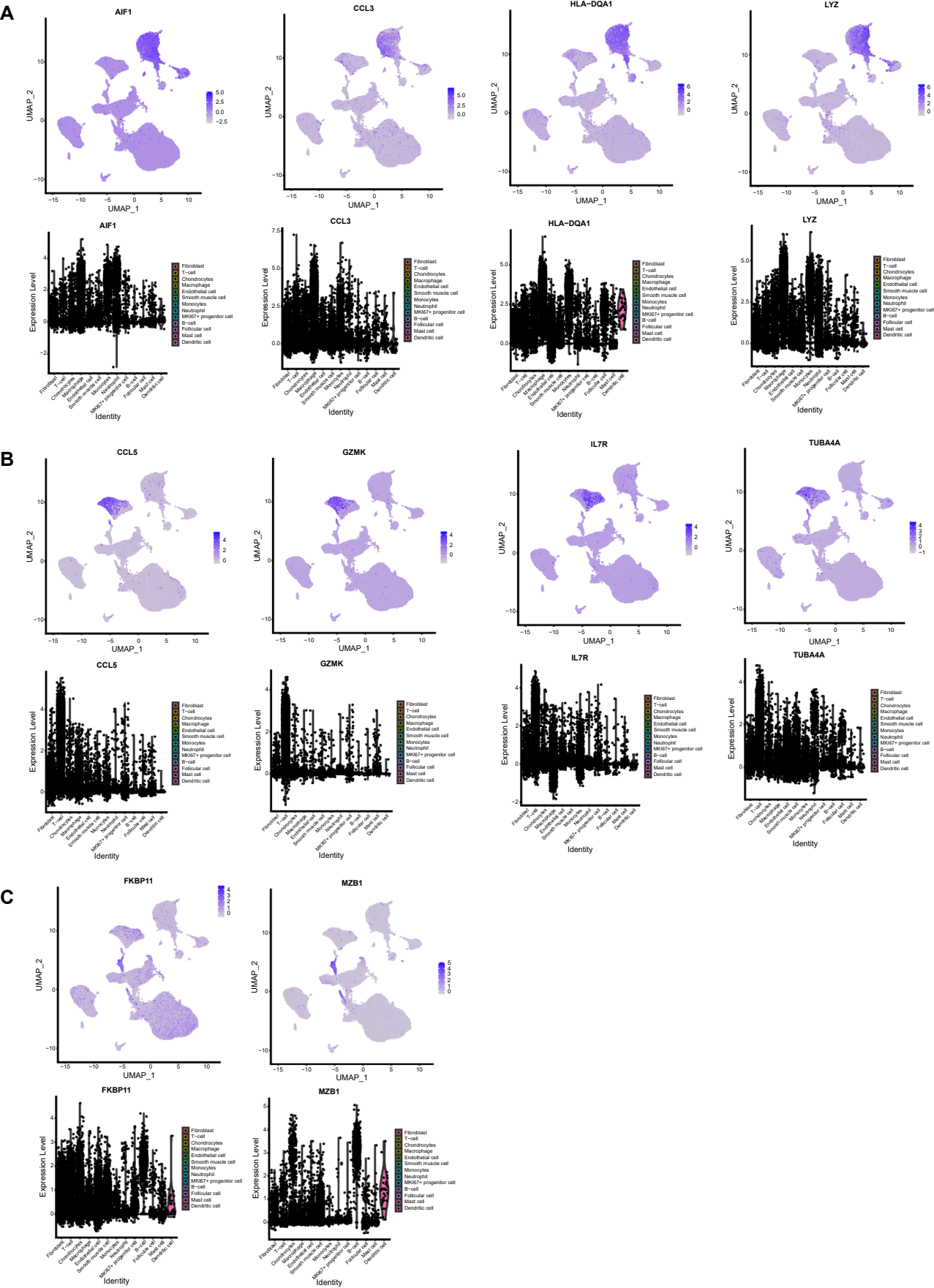

Supplement: Supplementary file 1 — Additional file 1: Figure 1 Imaging diagnosis results of the three patients in the first cohort. Figure 2 H&E staining and CD68 immunohistochemistry results of the OTB lesion tissue from (A) Patient 1, (B) Patient 2, and (C) Patient 3 in the first cohort. Figure 3 Quality control of single-cell sequencing data. Figure 4. Novel markers for macrophages/monocytes, T cells, and B cells and their clustering. Figure 5 Novel markers for specific myeloid cell subtypes and their clustering. Figure 6 B cell clusters in OTB PTs and ATs in the first cohort. Table 1. Detailed information of samples collected for scRNA-Seq analyses in the study. Table 2. Clinical characteristics of included participants in the second cohort. Table 3. Cell number and gene median statistics. Methods [file 10020_2024_886_MOESM1_ESM.zip › New folder/Sup Fig4-revised.pdf]

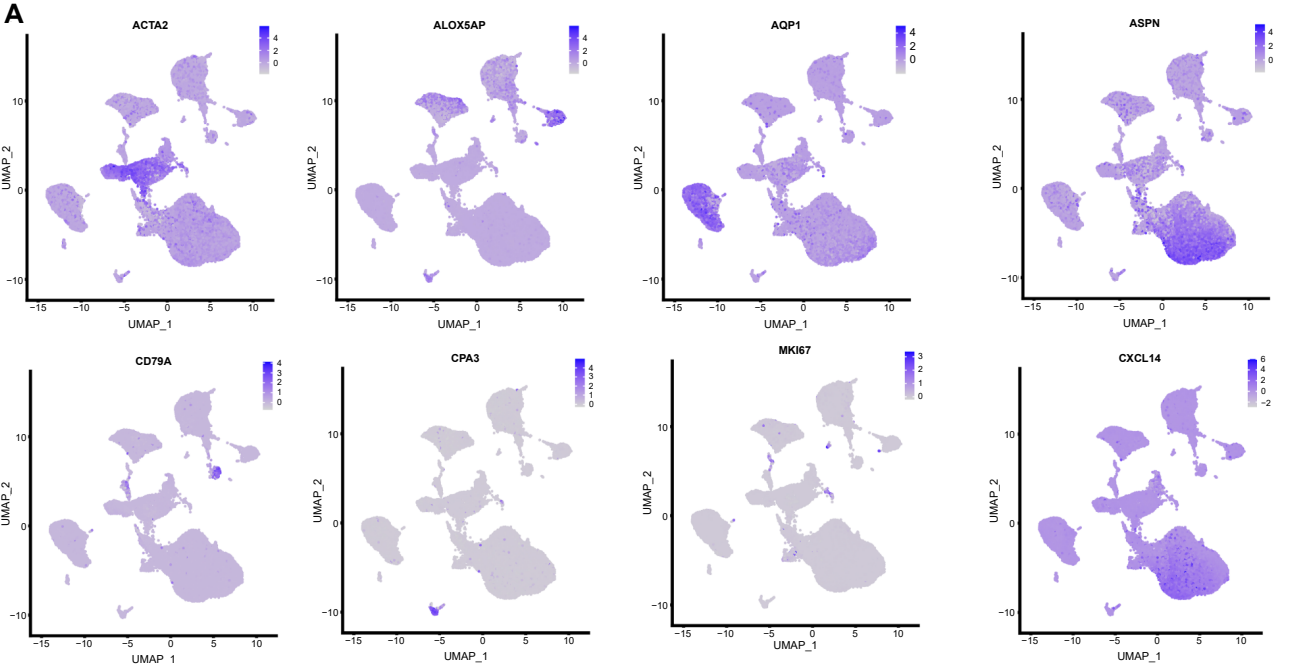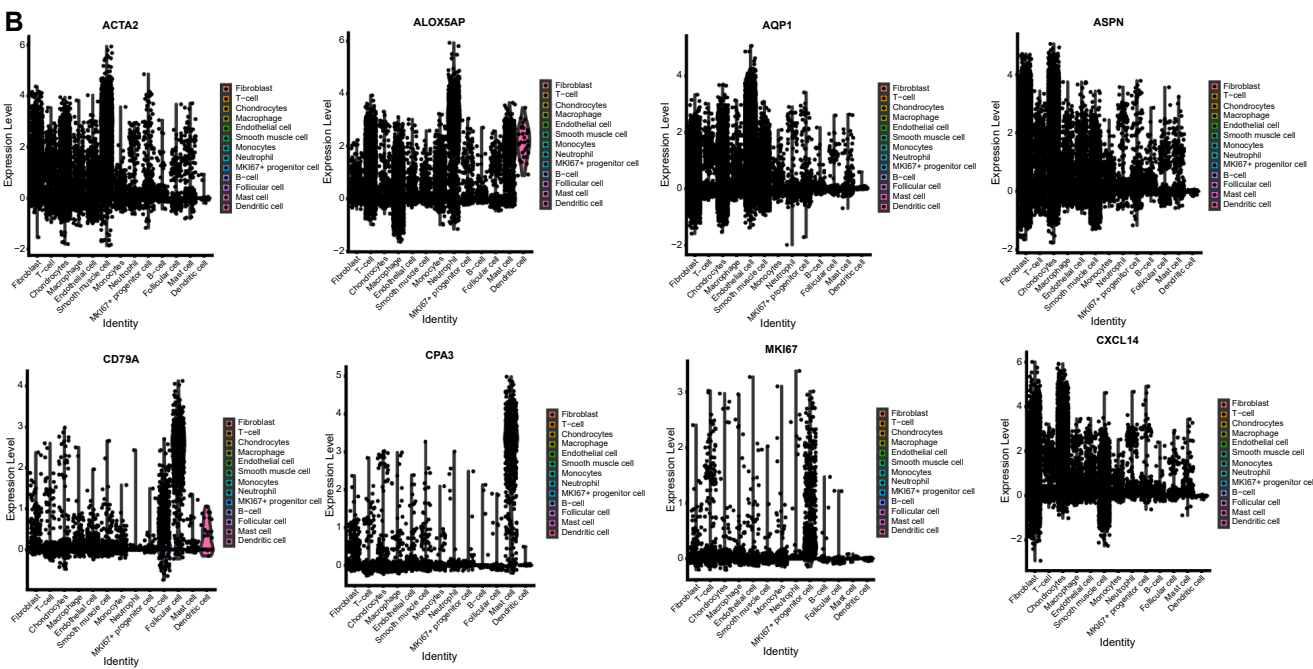

Supplement: Supplementary file 1 — Additional file 1: Figure 1 Imaging diagnosis results of the three patients in the first cohort. Figure 2 H&E staining and CD68 immunohistochemistry results of the OTB lesion tissue from (A) Patient 1, (B) Patient 2, and (C) Patient 3 in the first cohort. Figure 3 Quality control of single-cell sequencing data. Figure 4. Novel markers for macrophages/monocytes, T cells, and B cells and their clustering. Figure 5 Novel markers for specific myeloid cell subtypes and their clustering. Figure 6 B cell clusters in OTB PTs and ATs in the first cohort. Table 1. Detailed information of samples collected for scRNA-Seq analyses in the study. Table 2. Clinical characteristics of included participants in the second cohort. Table 3. Cell number and gene median statistics. Methods [file 10020_2024_886_MOESM1_ESM.zip › New folder/Sup Fig5-revised.pdf]

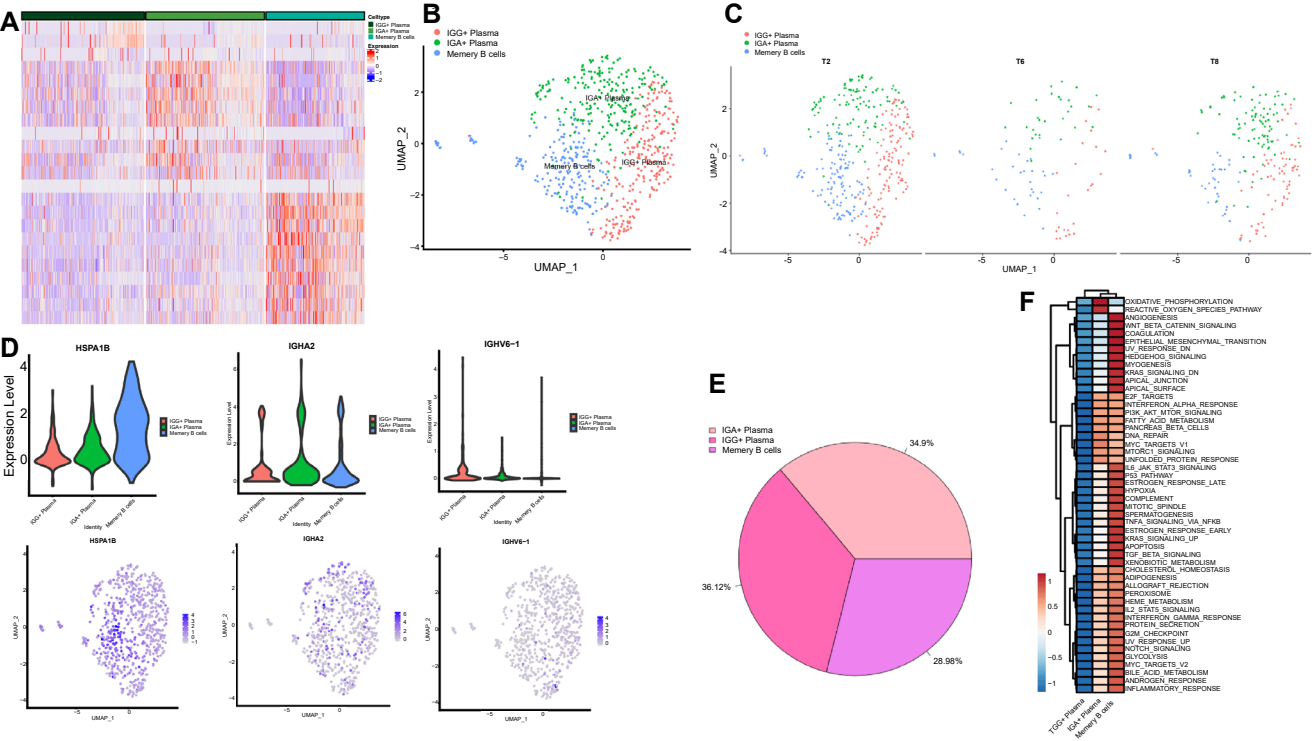

Supplement: Supplementary file 1 — Additional file 1: Figure 1 Imaging diagnosis results of the three patients in the first cohort. Figure 2 H&E staining and CD68 immunohistochemistry results of the OTB lesion tissue from (A) Patient 1, (B) Patient 2, and (C) Patient 3 in the first cohort. Figure 3 Quality control of single-cell sequencing data. Figure 4. Novel markers for macrophages/monocytes, T cells, and B cells and their clustering. Figure 5 Novel markers for specific myeloid cell subtypes and their clustering. Figure 6 B cell clusters in OTB PTs and ATs in the first cohort. Table 1. Detailed information of samples collected for scRNA-Seq analyses in the study. Table 2. Clinical characteristics of included participants in the second cohort. Table 3. Cell number and gene median statistics. Methods [file 10020_2024_886_MOESM1_ESM.zip › New folder/Sup Fig6.pdf]
